# Geminated Maxillary Lateral Incisor with Two Root Canals

**DOI:** 10.1155/2016/3759021

**Published:** 2016-12-29

**Authors:** Nayara Romano, Luis Eduardo Souza-Flamini, Isabela Lima Mendonça, Ricardo Gariba Silva, Antonio Miranda Cruz-Filho

**Affiliations:** Department of Restorative Dentistry, School of Dentistry of Ribeirão Preto, University of São Paulo, Ribeirão Preto, SP, Brazil

## Abstract

This paper reports a case of gemination in a maxillary lateral incisor with two root canals and crown-root dilaceration. A 16-year-old male patient was referred for endodontic treatment of the maxillary left lateral incisor and evaluation of esthetic and functional complaints in the anterior region. The patient reported trauma to the anterior primary teeth. There was no spontaneous pain, but the tooth responded positively to the vertical percussion test and negatively to the pulp vitality test. Clinical examination showed esthetic and functional alterations and normal periodontal tissues. CBCT imaging confirmed the suspicion of gemination and crown-root dilaceration and also revealed the presence of two root canals and periapical bone rarefaction. The root canals were instrumented with Reciproc R40 and 1% NaOCl irrigation and were filled by lateral condensation of gutta-percha and AH Plus sealer. The tooth was definitely restored with composite resin to recover esthetics. Continued follow-up over 6 months has shown absence of pain or clinical alterations as well as radiographic image suggestive of apical repair.

## 1. Introduction

Deep knowledge of the root canal system anatomy is critical to the success of endodontic therapy [1]. Anatomical complexities might interfere hindering the exploration, shaping, cleaning, and disinfection of root canals [2]. Maxillary lateral incisors normally have a single root and single canal [3]. However, morphological variations for these teeth include the presence of two [4, 5], three [6, 7], four [8], and even five canals [9], usually associated with the occurrence of traumatic stimuli during tooth development process [10]. Other morphological variations such as dens invagination [9, 11], radicular groove [12], and fusion [13] have also been reported.

Gemination is considered a rare developmental dental anomaly affecting the morphology of teeth. It is defined as a failed attempt at division of a single tooth germ by invagination, resulting in a tooth with a larger, incompletely separated crown having a single root and a single root canal [14]. There is no specific cause for the occurrence of this dental anomaly, which can be directly associated with genetic predisposition, racial characteristics, trauma to the primary dentition, and environmental factors, such as fetal exposure to alcohol, embryopathy by thalidomide, or hypervitaminosis A in pregnant women [15].

Although gemination can also occur in premolars and molars, it is more common in anterior teeth [16]. In most cases, the geminated tooth has a bifid crown with a single root and a single root canal and may cause aesthetic and functional impairments. Geminated and fused teeth have similar clinical appearance and clinical and radiographic examination is necessary for a differential diagnosis [17, 18].

In certain cases, two-dimensional imaging modalities, such as conventional and digital radiography, do not provide sufficient information for an accurate detection of anatomical variations and the use of more advanced auxiliary imagining resources is necessary. Imaging modalities that provide an undistorted three-dimensional vision of the tooth and surrounding structures should be used to improve the diagnostic potential. Cone-beam computed tomography (CBCT) provides three-dimensional images with high precision and sensitivity, offering a more detailed analysis of the case, a more adequate planning of root canal treatment, and guidance throughout the operative phase [19, 20].

This paper reports a case of gemination in a maxillary lateral incisor with two root canals and crown-root dilaceration.

## 2. Case Report

A 16-year-old male patient was referred to the clinic of our Department of Restorative Dentistry for evaluation and endodontic treatment of the maxillary left lateral incisor with main complaint of aesthetic and functional impairment.

Review of the patient's dental history revealed trauma to the anterior region of the maxilla around the age of 5 due to a fall. The traumatic injury caused intrusion of the primary maxillary left lateral incisor, which exfoliated after approximately 30 days. Orthodontic traction of the impacted permanent maxillary left lateral incisor was necessary at that time.

Clinical examination showed an amorphous, small, darkened crown and normal adjacent gingival tissues. There was no spontaneous pain, but the tooth responded positively to the vertical percussion test and negatively to the pulp vitality test.

**Figure 1:**
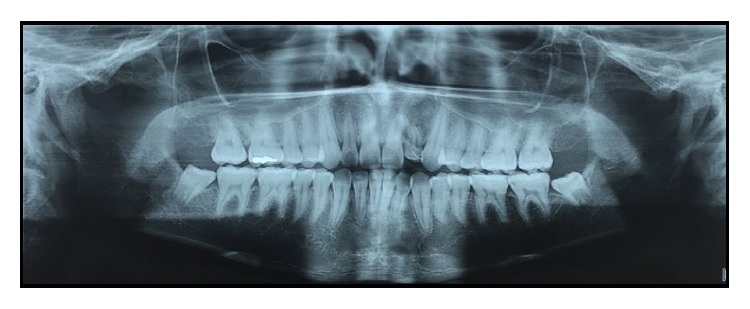
Panoramic radiograph.

**Figure 2:**
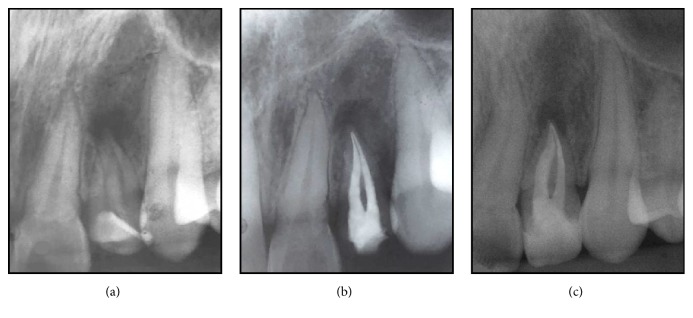
Operative imaging sequence. (a) Initial radiograph suggesting a geminated tooth; (b) final radiograph; (c) follow-up over 6 months' radiograph.

**Figure 3:**
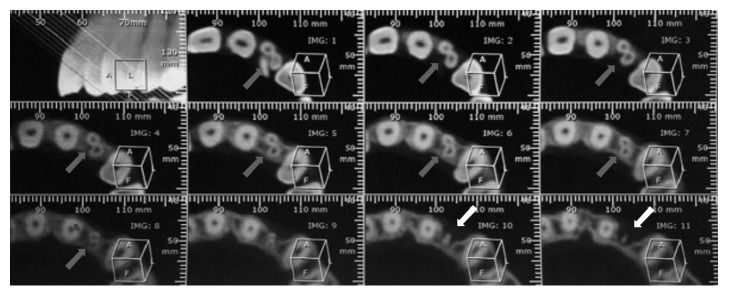
3D reconstruction of CBCT images with 0.25 mm axial slice thickness and 0.25 mm slice interval. Images 1–8 indicate the presence of two root canals in the maxillary left lateral incisor. Images 10 and 11 show apical rarefaction with destruction of the vestibular cortical bone.

Panoramic (Figure 1) and periapical (Figure 2(a)) radiographic examination suggested the existence of a developmental dental anomaly, with the presence of more than one root canal and apical rarefaction, as well as crown-root dilaceration. CBCT scanning was requested for a more detailed evaluation of the canal system and identification of dental structures (Figure 3) and confirmed gemination of the maxillary left lateral incisor and also revealed the presence of two root canals, one mesiobuccal and one distopalatal, and an apical hypodense lesion with destruction of the vestibular cortical bone (Figure 3, IMG 10 and 11).

Under local anesthesia and rubber dam isolation, access to the pulp chamber was achieved with round bur number 2 (KG Sorensen, São Paulo, SP, Brazil) and Endo-Z bur (Dentsply Maillefer, Ballaigues, Switzerland). Because of the difficulty in locating the canal openings, an ultrasonic insert (E3D; Helse Indústria e Comércio, Santa Rosa de Viterbo, SP, Brazil) was used to remove dentin tissue until finding the entrances of the mesiobuccal and distopalatal canals. The pulp chamber was flooded with 1% NaOCl solution and the canals were explored with a size 10 K file. In both canals, the working length was determined at 18 mm using an electronic apex locator (Root ZX mini, J Morita Mfg Corp., Japan). The canals were instrumented with R40 file of the Reciproc system (Reciproc, VDW, Germany) under irrigation with 1% NaOCl. Root canal treatment was carried out in two sessions. At the end of the first session, a calcium hydroxide paste was used as an intracanal medication and restorative glass-ionomer cement was used as temporary restoration. In the second session, after 15 days, the intracanal medication was removed and the canals were filled with 17% EDTA for 3 minutes for smear layer removal, irrigated again with 1% NaOCl, dried with absorbent paper points, and filled by lateral condensation of gutta-percha and AH Plus sealer (Dentsply/De Trey, Konstanz, Germany) (Figure 2(b)). The patient returned after 30 days for evaluation. Clinically, the tooth presented no painful symptomatology or discomfort and the adjacent gingival tissue was normal. Radiographic examination showed no signs of failure in root canal filling or periapical lesions. The tooth crown was definitely restored with composite resin (Charisma A3, Heraeus Kulzer GmbH, Hanau, Germany) to recover esthetics and function.

Continued follow-up over 6 months has shown a successful outcome with absence of pain, no clinical alterations, and radiographic image suggestive of apical repair (Figure 2(c)).

## 3. Discussion

An accurate diagnosis and the precise determination of the number of root canals are mandatory for the success of endodontic treatment. Overlooking dental anomalies and anatomical variations can have a negative impact on treatment outcome, with persistence or exacerbation of preexisting apical periodontitis [21].

Radiographic examination is an important part in diagnosis and treatment planning [22]. Periapical radiographs with variations of angulation can be obtained to increase their accuracy in identifying anatomical anomalies [23]. However, conventional radiography offers limited information because it provides a two-dimensional image and there is a possibility of distortion and superimposition of structures. Computed tomography provides three-dimensional images, reproducing the structures more precisely and allowing a more accurate diagnosis [24]. In the present case, CBCT was essential to confirm the diagnosis of gemination as well as to determine the exact number and the precise location of the canals in order to have a safer access to the canal system during shaping, cleaning, and filling procedures. It should also be mentioned that the use of a surgical microscope is a widely employed clinical resource for the cases in which difficulty is experienced in the localization of root canals [25].

The maxillary lateral incisor usually has a single root and a single canal [3, 26]. However, cases of maxillary lateral incisors with more than one canal and separate roots have been reported [4, 8, 27]. Variations in the number of canals are associated with dental anomalies or intrusive trauma to the primary teeth during the development of the permanent successors [27, 28].

Gemination is less prevalent in the permanent dentition than in the primary dentition, affecting mainly maxillary incisors and canines [29]. The incidence of gemination in permanent teeth has been shown to range from 0.1% to 1% [30]. This dental anomaly has a direct impact on the development of the dentition, altering the mesiodistal dimension of the anomalous tooth and its alignment in the dental arch [31]. In the present case, the remainder of the crown showed a deviation relative to the long axis of the root, which was diagnosed as crown-root dilaceration. Dilaceration can affect any region of the tooth from the crown to the root apex. Crown dilaceration in permanent teeth is more frequent in cases with history of avulsion or intrusion of the primary predecessor. Traumatic dental injuries in the primary dentition occur more often in children between 1.5 and 3.5 years old [22, 32].

The etiology of gemination is not clear, but it is known to be associated with trauma to the primary dentition during the development of the permanent tooth germ. Some authors have also claimed that gemination could be the result of the interaction of hereditary genetic variations and environmental factors [33, 34]. In the present case, the diagnosis of gemination was established based on patient's dental history, CBCT imaging, and the concept of gemination reported in the literature.

Gemination can often be confused with fusion, which is a developmental dental anomaly characterized by the union of two adjacent tooth germs, resulting in a reduction in the number of teeth in affected arch. The differential diagnosis is usually made by counting the number of teeth in the arch, which is not altered in the cases of gemination [18, 35].

Developmental dental anomalies like gemination can cause functional, orthodontic, endodontic, and esthetic impairments and represent a challenge for dentists because, in most cases, a multidisciplinary approach is required to obtain the best treatment and a successful outcome [36].

## 4. Conclusion

Although it is considered a rare developmental dental anomaly with low prevalence, the occurrence of gemination deserves attention in clinical practice. Knowledge of the literature-based definition of this anomaly, review of dental history, and the use of accurate imaging resources, such as CBCT, are essential for a correct diagnosis and establishment of an adequate treatment plan in geminated teeth.
